# Supramolecular Diiodine-Bromostannate(IV) Complexes: Narrow Bandgap Semiconductors

**DOI:** 10.3390/molecules27123859

**Published:** 2022-06-16

**Authors:** Nikita A. Korobeynikov, Andrey N. Usoltsev, Pavel A. Abramov, Maxim N. Sokolov, Sergey A. Adonin

**Affiliations:** Nikolaev Institute of Inorganic Chemistry SB RAS, 630090 Novosibirsk, Russia; 27041998rus@gmail.com (N.A.K.); usoltsev@niic.nsc.ru (A.N.U.); abramov@niic.nsc.ru (P.A.A.); caesar@niic.nsc.ru (M.N.S.)

**Keywords:** polyhalogens, halogen bonding, non-covalent interactions, tin, halide complexes, iodine

## Abstract

Three supramolecular bromostannates(IV) with “trapped” diiodine molecules, Cat_2_{[SnBr_6_](I_2_)} (Cat = Me_4_N^+^ (**1**), 1-MePy^+^ (**2**) and 4-MePyH (**3**)), were synthesized. In all cases, I_2_ linkers are connected with bromide ligands via halogen···halogen non-covalent interactions. Articles **1–3** were studied using Raman spectroscopy, thermogravimetric analysis, and diffuse reflectance spectroscopy. The latter indicates that **1–3** are narrow band gap semiconductors.

## 1. Introduction

Metal halide complexes, or halometalates, represent the class of coordination compounds which, apart from fundamental interest (in particular, related to structural chemistry), attracts attention due to numerous areas of materials science where they can be applied. Those include design of ferroelectric and ferroelastic materials [1,2,3,4,5], photocatalysis [6,7,8], luminescence [9,10,11,12,13,14,15], photochromism [16,17], etc. However, the most prominent area is photovoltaics. Since it was discovered that 3D perovskite-type iodoplumbates(II) can be used as light absorbers in solar cells, this area has experienced very rapid growth [18,19,20,21,22,23,24,25,26,27] (according to Scopus data, the number of papers on this topic dramatically increases annually). In the last few years, there appear many works about the use of iodometalates of p-block metals other than lead (this is driven mostly by environmental considerations). Among the elements considered for this purpose, there are bismuth, tin, antimony, etc. [28]. Yet, performance of solar cells based thereupon is significantly lower than for iodoplumbate(II) derivatives but, at the same time, it was found that iodometalates(III) (M = Sb, Bi) can serve as a component of highly efficient photodetectors [29,30,31,32,33,34]. This fact strongly inspires further research in this field.

It is commonly assumed that the main disadvantage of halometalates other than Pb(II) and Sn(II) derivatives in the course of photovoltaic applications is their stereochemistry. While iodoplumbates(II) and –stannates(II) can form isotropic, covalently-bonded 3D structures (perovskite-type), this is impossible for Sb(III) and Bi(III), which usually form discrete, 1D or, rarely, 2D anions [35]. For M(IV) halide complexes, it is even more challenging since they most commonly appear as mononuclear [MX_6_]^2−^ anions. A possible strategy to overcome this problem is the use of supramolecular approaches (increasing of dimensionality via non-covalent interactions in a solid state). One of such ideas presented recently is the use of supramolecular polyhalogen-halometalate hybrids. Incorporation of di- or polyiodide units into a structure commonly yields a narrower optical band gap of resulting compounds [36,37] and the assembly of 1D, 2D, or even 3D associates via halogen bond [38]. Recent examples of diiodo-bromoantimonates(III) and –bromotellurates(IV) demonstrate [39,40] that such hybrids indeed can serve as components of photodetectors. Therefore, a search of new compounds belonging to this family is well justified.

Although tin(IV) halide complexes feature stereochemistry almost identical to Te(IV), corresponding polyiodo-halometalates have yet been unknown. In this work, we present the first examples of this class: Cat_2_{[SnBr_6_](I_2_)} (cat = Me4N^+^ (**1**), 1-MePy^+^ (**2**) and 4-MePyH^+^ (**3**). In addition to the structural studies, we hereby report their thermal stability, optical properties, and Raman spectra.

## 2. Experimental Part

All reagents were obtained from commercial sources and used and purchased. 1-methylpyridinium iodide (1-MePyI) was prepared by reaction of pyridine and 1.1× excess of methyl iodide. Elemental analysis was performed on a Euro NA 3000 Elemental analyzer (EuroVector, Pavia, Italy). In all cases, concentrated aqueous HBr was used.

### 2.1. Synthesis of 1

An amount of 60 mg (0.22 mmol) of SnBr_2_ was dissolved in 3 mL of HBr at 70 °C. Then, 100 mg (0.43 mmol) of I_2_ were added. After 15 min, a solution of Me_4_Br in 2 mL of HBr was added. The hot mixture was filtered, slowly cooled to r.t., and then to 5 °C. Within 6 h, dark cherry-red crystals of **1** were formed. The yield was 65%. Element analysis calculated for C_8_H_24_N_2_SnBr_6_I_2_: C 9.64; H 2.43; N 2.81. Found: C 9.74; H 2.51; N 2.90.

### 2.2. Synthesis of 2

An amount of 192 mg (0.87 mmol) of 1-MePyI was dissolved in 4 mL of water and mixed with a stoichiometric amount of AgNO_3_ with stirring. After filtering off the formed AgI, the resulting solution was evaporated and the precipitate was dissolved in 4 mL of HBr. The mixture was heated to 70 °C. After that, 120 mg (0.43 mmol) SnBr_2_ and 220 mg (0.87 mmol) of I_2_ were added. After cooling the solution, it was kept at 5 °C for one day, resulting in dark cherry-red crystals of **2**. Yield: 67%. Element analysis calculated for C_12_H_16_N_2_SnBr_6_I_2_: C 13.91; H 1.56; N 2.70. Found: C 14.00; H 1.61; N 2.78.

### 2.3. Synthesis of 3

An amount of 120 mg (0.43 mmol) of SnBr_2_ was dissolved in 4 mL of HBr_._ The solution was heated to 70 °C; 220 mg (0.87 mmol) of I_2_ was added and the mixture was stirred for 30 min. After that, 85 μL (0.87 mmol) of 4-MePy were added. The mixture was cooled to r.t., then to 5 °C. After 7 h, dark cherry-red crystals of **3** formed. The estimated yield was 63%. Element analysis calculated for C_12_H_16_N_2_SnBr_6_: C 18.43; H 2.06; N 3.58. Found: C 18.38; H 2.13; N 3.57.

### 2.4. X-ray Diffractometry

Crystallographic data and refinement details for **1–3** are given in Table S1 (SI). The diffraction data were collected on a New Xcalibur (Agilent Technologies, Santa Clara, CA, USA) diffractometer with MoKα radiation (λ = 0.71073) by doing φ scans of narrow (0.5°) frames at 150 K. Absorption correction was done empirically using SCALE3 ABSPACK (CrysAlisPro, Agilent Technologies, Version 1.171.37.35 (release 13-08-2014 CrysAlis171.NET) (compiled 13 August 2014, 18:06:01)).

Structures were solved by SHELXT [41] and refined by full-matrix least-squares treatment against |F|^2^ in anisotropic approximation with SHELX 2014/7 [42] in ShelXle program [43]. H-atoms were refined in the geometrically calculated positions. The main geometrical parameters are summarized in Table S2.

The structure of 1 was refined in *I*4_1_cd as an inversion twin with BASF 0.48. PLATON did not suggest any symmetry change. Attempts to solve the structure in *I*4_1_/acd or P4_2_nm space groups did not give any improvements. One position of highly disordered CH_3_ group around N3 was not refined due to the absence of q-peaks with right geometry. Splitting and refinement of C-atoms generated based on found positions were unstable. The occupancy of N3 was 100%. The refinement composition was C_7.75_H_23.25_Br_6_I_2_N_2_Sn. The elemental analysis data confirmed the complex composition as C_8_H_24_Br_6_I_2_N_2_Sn. According to the electron density distribution, the second position of [SnBr_6_]^2−^ octahedral unit with ≈8% occupancy has been found (see SI for details).

The crystallographic data have been deposed in the Cambridge Crystallographic Data Centre under the deposition codes CCDC 2170497-2170499.

### 2.5. Raman Spectroscopy

Raman spectra were collected using a LabRAM HR Evolution (Horiba) spectrometer with the excitation by the 633 nm line of the He-Ne laser. The spectra at room temperatures were obtained in the backscattering geometry with a Raman microscope. The laser beam was focused to a diameter of 2 μm using a LMPlan FL 50×/0.50 Olympus objective. The spectral resolution was 0.7 cm^−1^. The laser power on the sample surface was about 0.03 mW.

### 2.6. Diffuse Reflectance Spectroscopy

Diffuse reflectance spectra were measured on a setup which consisted of a Kolibri-2 spectrometer (VMK Optoelektronica, Novosibirsk, Russia), fiber optic cable QR-400-7 (Ocean Optics, Rochester, NY, USA), and deuterium–tungsten lamp AvaLight-DHS (Avantes, Apeldoorn, The Netherlands). The reference of 100% reflectance was BaSO4 powder. The spectra were recorded five times in the wavelength interval of 300–1000 nm and then averaged to reduce the random error.

### 2.7. Thermogravimetric Analysis (TGA)

TGA were carried out on a TG 209 F1 Iris thermobalance (NETZSCH, Selb, Germany). The measurements were made in a helium flow in the temperature range of 30–450 °C using the heating rate of 10 °C min^−1^, the gas flow rate of 60 mL min^−1^, and open Al crucibles.

### 2.8. Powder X-ray Diffractometry (PXRD)

XRD analysis of polycrystals was performed on a Shimadzu XRD-7000 diffractometer (CuK-alpha radiation, Ni—filter, linear One Sight detector, 5–50° 2θ range, 0.0143° 2θ step, 2 s per step). Plotting of PXRD patterns and data treatment was performed using X’Pert Plus software (see SI).

## 3. Results and Discussion

Synthesizing complexes **1–3**, we used the same “straightforward” approach [39,40] as we extensively applied earlier for Bi(III), Sb(V), and Te(IV) complexes: a solution containing anionic bromometalate complex anions was mixed with I_2_ and then with the salt of the organic cation. The choice of the latter is known to play the most important role in halometalate chemistry since the nature of the cation has a very strong influence on the system of non-covalent interactions in solid state and, therefore, it affects the assembly of polynuclear anions. However, very little is known about rational principles of precursor selection—in other words, screening is yet the most common strategy in halometalate chemistry. On this reason, we chose highly available cations which we already used in our earlier works on polyhalogen-halometalates.

The XRD data indicate that, like in halotellurates(IV), all isolated complexes are built out of mononuclear [SnBr_6_]^2−^ anions. The Sn-Br bond lengths are 2.587–2.645 Å in **1**, 2.563–2.627 Å in **2**, and 2.563–2.684 Å in **3**, respectively. The composition of the polyhalogeno-anionic part in all cases is identical: {[SnBr_6_](I_2_)}. The I···I bonds in **1–3** are 2.692–2.695, 2.703, and 2.686 Å, respectively.

**Figure 1:**
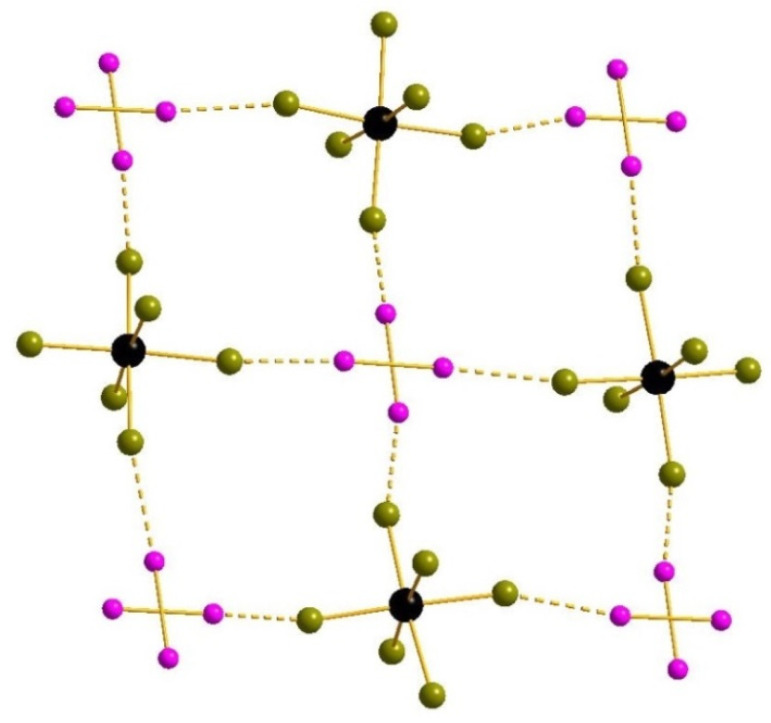
Supramolecular associates built of [SnBr_6_]^2−^ and I_2_ units in the structure of **1**. Here and below: Sn black, Br olive-green, I purple, I···Br interactions dashed. All positions of disordered I_2_ fragments are shown.

In **1**, the I_2_ units are disordered over two positions with 0.985:0.015 occupancies. The [SnBr_6_]^2−^ units form non-covalent interactions with I_2_ (for most populated I_2_ positions, corresponding Br···I are 3.201–3.203 Å which is less than the sum of corresponding Bondi’s van der Waals radii (3.81 Å [44,45]). The I-I-Br angles are 170.57–173.88°. The superposition of {[SnBr_6_](I_2_)} chains can be considered as a pseudo-2D substructure (Figure 1). The positional disorder of [SnBr_6_]^2−^ over two closed positions (0.92/0.08 occupancies) was found in the crystal structure of **1** (Figure S3, SI). Such complex anions interact with I_2_ molecules, producing layers where both components follow 44 plane net topology (Figure S4). Such layers stack together in ABABA… manner (Figure S5) with shifting in a [110] crystal direction. Both [SnBr_6_]^2−^ and I_2_ centers of gravity follow bcc sublattices. TMA cations occupy voids between the above-mentioned layers (Figure S6). There are three types of TMA cations in the unit cell (Figure S7): the first and second types occupy special positions and the third type occupies common positions. Cations of the latter type have non-linear periodicity.

**Figure 2:**
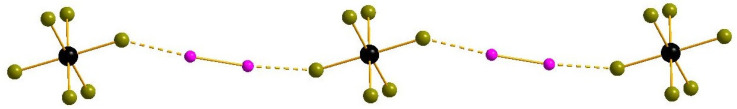
Linear {[SnBr_6_](I_2_)}^2n−^ associates in the structures of **2** and **3**.

In both **2** and **3**, I_2_ and [SnBr_6_]^2−^ structural units form linear supramolecular chains (Figure 2). The I···Br distances in **2** and **3** are 3.219 and 3.412–3.432 Å, the Sn-Br-I and Br-I-I angles are 143.46 and 173.03 in **2**, 160.00–167.09 and 162.67–170.18°, respectively.

In **2**, there are two types of above-mentioned 1D chains running perpendicular to each other along [b − a] and [b + a] directions, respectively (Figure S7, SI). In the crystal, packing such 1D structures can be combined into pseudo layers located in the [110] crystal direction. These layers stack together with AABBAA… topology. 1-methylpyridinium cations are located in the space between the above-mentioned 1D associates. In **3**, [SnBr_6_]^2−^ and I_2_ form chains running along [010] the crystal direction. In the crystal packing (Figure S8), such associates can be combined into pseudo layers located in [110] the crystal direction. These layers stack together, producing ABABA… topology. 4-methylpyridinium cations are located in the space between the above-mentioned 1D structures. Hence, changing of the Me-group position strongly affects the crystal packing topology of isolated compounds.

**Figure 3:**
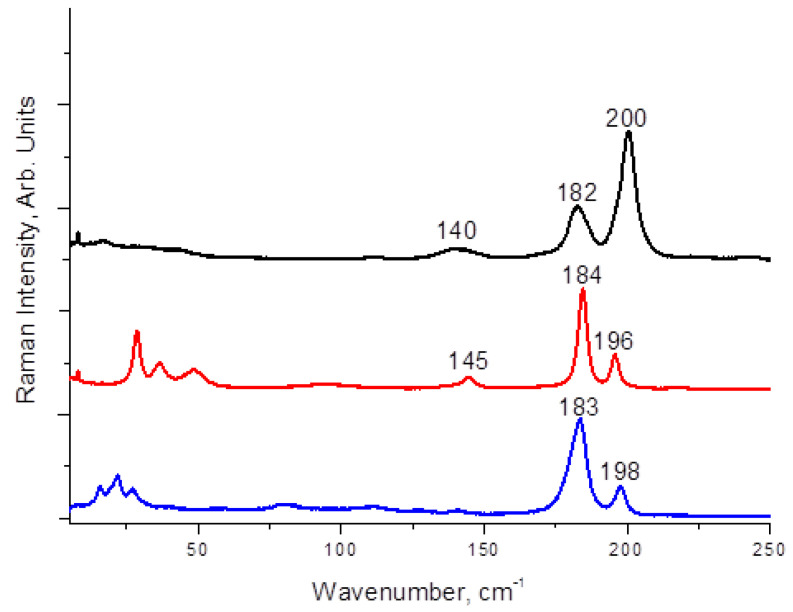
Raman spectra of **1** (black), red (**2**), and blue (**3**).

Complex **3** demonstrates poor stability while being isolated from I_2_-containing mother liquor. It decomposes within a few dozen minutes, losing incorporated I_2_ (elemental analysis for the residue corresponds to “diiodine-free” (4-MePyH)_2_[SnBr_6_]). Both **1** and **2** are stable and they were isolated as single phases, as follows from PXRD data (see SI, Figures S1 and S2). Nevertheless, we succeeded in recording of Raman spectra for the whole series of samples. Results are presented on Figure 3. It can be noticed that in each case, there are two bands within the 180–200 cm^−1^ region where I_2_ has a highly characteristic band in diiodo-halometalates [46]. Most likely, the bands at 196–200 cm^−1^ are related to I_2_ while those at 182–184 correspond to [SnBr_6_]^2−^ vibrations [47] (as well as all bands at wavelengths < 150 cm^−1^; these data agree well with our previous work [48]).

**Figure 4:**
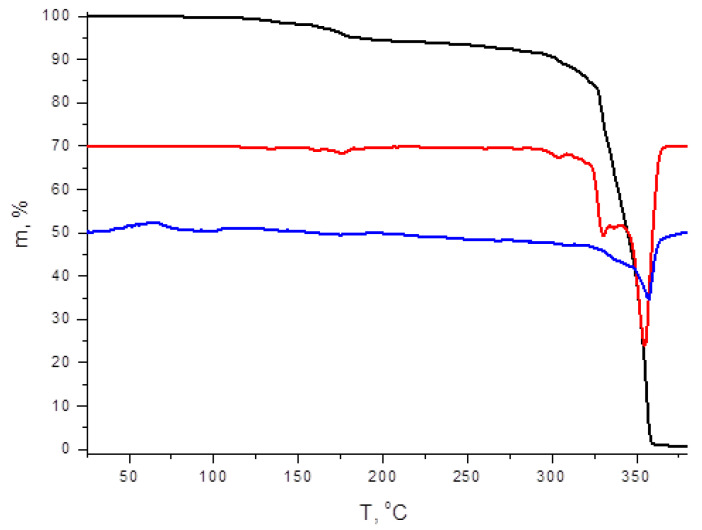
TG, DTG, and DTA curves of **1**.

**Figure 5:**
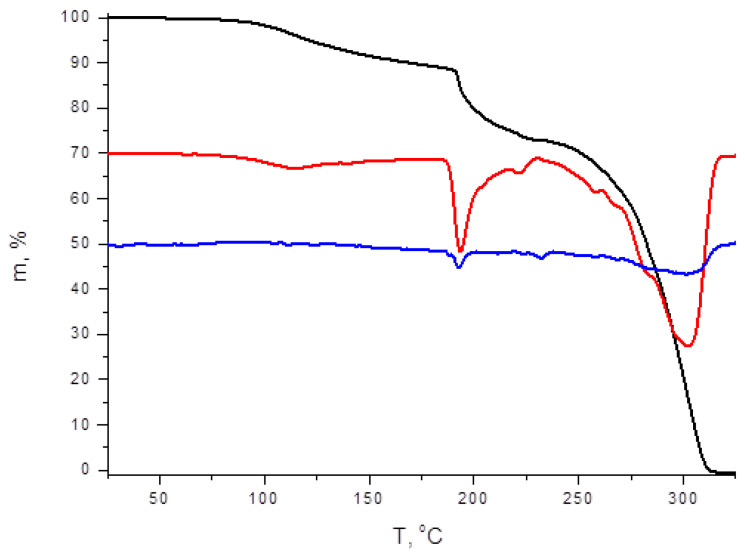
TG, DTG, and DTA curves of **2**.

As follows from TGA data, **1** and **2** demonstrate different thermal stability—decomposition corresponding to continuous loss of diiodine occurs at >110 and >90 °C, respectively (Figure 4 and Figure 5). These results agree well with our observations made for diiodo-bromotellurates(IV) [40]: depending on the nature of the organic cation and, therefore, on the system of EH···X interactions it forms in solid state with halide ligands and polyhalogeno units, stability can be dramatically diverse.

**Figure 6:**
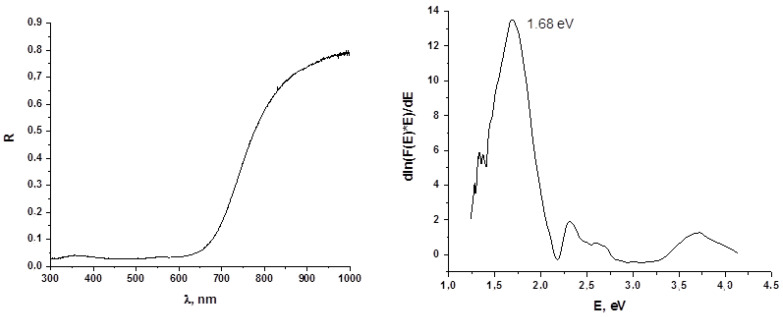
Diffuse reflectance spectrum (**left**) and optical band gap determination (Tauc coordinates, **right**) for **1**.

**Figure 7:**
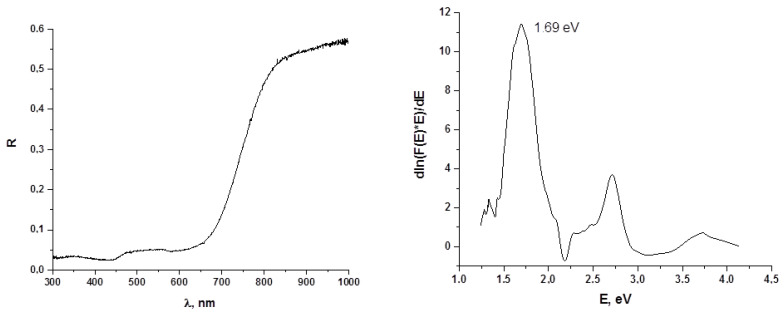
Diffuse reflectance spectrum (**left**) and optical band gap determination (Tauc coordinates, **right**) for **2**.

Diffuse reflectance spectra of **1** and **2** are presented in Figure 6 and Figure 7. The optical band gaps for these two complexes are 1.68 and 1.69 eV, respectively, which is more than for similar diiodo-bromotellurates described by us earlier (1.41–1.74, with average 1.55 eV [40]). The overall features of these spectra are quite common for p-block halometalates [49,50].

## 4. Conclusions

This work demonstrates that the general strategy utilized in preparation of polyhalogen-halometalates, designed and widely applied by us for synthesis of Bi(III), Te(IV), and Sb(V) complexes, as well as dichloro-chlorostannates(IV) and –plumbates(IV) [51], works very well for diiodo-bromostannates(IV). We assume the existence of such hybrids of other metals commonly forming mononuclear halometalate anions [MX_6_]^2−^, such as Zr, Hf, Ir, etc. Corresponding experiments are underway in our group.

## Data Availability

Not applicable.

## Supplementary Materials

The following supporting information can be downloaded at: https://www.mdpi.com/article/10.3390/molecules27123859/s1, Figure S1: Calculated (black) and experimental (red) PXRD patterns of **1**; Figure S2: Calculated (black) and experimental (red) PXRD patterns of **2**; Figure S3: Positional disordering of [SnBr_6_]^2−^ octahedron over two closed positions with 0.92 (red) and 0.08 (blue) occupancies; Figure S4: Structure of the layer formed by [SnBr_6_]^2−^ and I2 units; Figure S5: Layered crystal packing of **1**; Figure S6: Positions of TMA+ cations in the void between {[SnBr_6_]^2−^ + I2} nets; Figure S7: Crystal packing of **2**; Figure S8. Crystal packing of **3**; Table S1: Experimental details; Table S2: Selected geometric parameters (Å).

## Sample Availability

Not available.
