# Supplementary material for: Supramolecular Diiodine-Bromostannate(IV) Complexes: Narrow Bandgap Semiconductors
Source: Molecules. 2022 Jun 16;27(12):3859. doi: 10.3390/molecules27123859 (PMC9229721; doi:10.3390/molecules27123859)

checkCIF/PLATON report

Structure factors have been supplied for datablock(s) 1, 2, 3

THIS REPORT IS FOR GUIDANCE ONLY. IF USED AS PART OF A REVIEW PROCEDURE FOR PUBLICATION, IT SHOULD NOT REPLACE THE EXPERTISE OF AN EXPERIENCED CRYSTALLOGRAPHIC REFEREE.

No syntax errors found. CIF dictionary Interpreting this report

Datablock: 1

Bond precision: N- C = 0.0145 A Wavelength=0.71073

Cell:	a=19.4249(4)	b=19.4249(4)	c=26.0612(8)
	alpha=90	beta=90	gamma=90
Temperature:	150 K		

	Calculated	Reported
Volume	9833.6(5)	9833.6(5)
Space group	I 41 c d	I 41 c d
Hall group	I 4bw -2c	I 4bw -2c
Moiety formula	4(Br6 I0.03 Sn), C7 H21 N2, 6(C4 H12 N), 7.88(I)	?
Sum formula	C31 H93 Br24 I8 N8 Sn4	C7.75 H23.25 Br6 I2 N2 Sn
Mr	3985.77	996.48
Dx,g cm-3	2.692	2.692
Z	4	16
Mu (mm-1)	13.295	13.295
F000	7196.0	7196.0
F000'	7149.18	
h,k,lmax	26,26,35	25,24,34
Nref	6548[3346]	5187
Tmin,Tmax	0.288,0.394	0.661,1.000
Tmin'	0.243	

Correction method= # Reported T Limits: Tmin=0.661 Tmax=1.000
AbsCorr = MULTI-SCAN

Data completeness= 1.55/0.79 Theta(max)= 28.994

R(reflections)= 0.0320(4466) wR2(reflections)= 0.0757(5187)

S = 1.074 Npar= 188

The following ALERTS were generated. Each ALERT has the format
test-name_ALERT_alert-type_alert-level.
Click on the hyperlinks for more details of the test.

Alert level C

```
STRVA01_ALERT_4_C          Flack test results are ambiguous.
           From the CIF: _refine_ls_abs_structure_Flack    0.483
           From the CIF: _refine_ls_abs_structure_Flack_su    0.014
PLAT048_ALERT_1_C MoietyFormula Not Given (or Incomplete) ........     Please Check
PLAT094_ALERT_2_C Ratio of Maximum / Minimum Residual Density ....       2.03 Report
PLAT242_ALERT_2_C Low    'MainMol' Ueq as Compared to Neighbors of        Sn1 Check
PLAT242_ALERT_2_C Low    'MainMol' Ueq as Compared to Neighbors of         N3 Check
PLAT242_ALERT_2_C Low    'MainMol' Ueq as Compared to Neighbors of         N1 Check
PLAT242_ALERT_2_C Low    'MainMol' Ueq as Compared to Neighbors of         N2 Check
PLAT911_ALERT_3_C Missing FCF Refl Between Thmin & STh/L=    0.600          2 Report
PLAT934_ALERT_3_C Number of (Iobs-Icalc)/Sigma(W) > 10 Outliers ..          1 Check
PLAT971_ALERT_2_C Check Calcd Resid. Dens.  0.78Ang From Sn1             1.80 eA-3
```

Alert level G

```
PLAT004_ALERT_5_G Polymeric Structure Found with Maximum Dimension          2 Info
PLAT045_ALERT_1_G Calculated and Reported Z Differ by a Factor ...         0. Check
PLAT300_ALERT_4_G Atom Site Occupancy of I3         Constrained at      0.015 Check
PLAT300_ALERT_4_G Atom Site Occupancy of I4         Constrained at      0.015 Check
PLAT300_ALERT_4_G Atom Site Occupancy of C7         Constrained at        0.5 Check
PLAT300_ALERT_4_G Atom Site Occupancy of C8         Constrained at       0.25 Check
PLAT300_ALERT_4_G Atom Site Occupancy of C11        Constrained at       0.25 Check
PLAT300_ALERT_4_G Atom Site Occupancy of C12        Constrained at       0.25 Check
PLAT300_ALERT_4_G Atom Site Occupancy of C13        Constrained at       0.25 Check
PLAT300_ALERT_4_G Atom Site Occupancy of C14        Constrained at       0.25 Check
PLAT300_ALERT_4_G Atom Site Occupancy of H7A        Constrained at        0.5 Check
PLAT300_ALERT_4_G Atom Site Occupancy of H7B        Constrained at        0.5 Check
PLAT300_ALERT_4_G Atom Site Occupancy of H7C        Constrained at        0.5 Check
PLAT300_ALERT_4_G Atom Site Occupancy of H8A        Constrained at       0.25 Check
PLAT300_ALERT_4_G Atom Site Occupancy of H8B        Constrained at       0.25 Check
PLAT300_ALERT_4_G Atom Site Occupancy of H8C        Constrained at       0.25 Check
PLAT300_ALERT_4_G Atom Site Occupancy of H11A       Constrained at       0.25 Check
PLAT300_ALERT_4_G Atom Site Occupancy of H11B       Constrained at       0.25 Check
PLAT300_ALERT_4_G Atom Site Occupancy of H11C       Constrained at       0.25 Check
PLAT300_ALERT_4_G Atom Site Occupancy of H12A       Constrained at       0.25 Check
PLAT300_ALERT_4_G Atom Site Occupancy of H12B       Constrained at       0.25 Check
PLAT300_ALERT_4_G Atom Site Occupancy of H12C       Constrained at       0.25 Check
PLAT300_ALERT_4_G Atom Site Occupancy of H13A       Constrained at       0.25 Check
PLAT300_ALERT_4_G Atom Site Occupancy of H13B       Constrained at       0.25 Check
PLAT300_ALERT_4_G Atom Site Occupancy of H13C       Constrained at       0.25 Check
PLAT300_ALERT_4_G Atom Site Occupancy of H14A       Constrained at       0.25 Check
PLAT300_ALERT_4_G Atom Site Occupancy of H14B       Constrained at       0.25 Check
PLAT300_ALERT_4_G Atom Site Occupancy of H14C       Constrained at       0.25 Check
PLAT300_ALERT_4_G Atom Site Occupancy of I1         Constrained at      0.985 Check
PLAT300_ALERT_4_G Atom Site Occupancy of I2         Constrained at      0.985 Check
PLAT301_ALERT_3_G Main Residue  Disorder ..............(Resd  1  )         0% Note
PLAT301_ALERT_3_G Main Residue  Disorder ..............(Resd  2  )        78% Note
PLAT302_ALERT_4_G Anion/Solvent/Minor-Residue Disorder (Resd  5  )       100% Note
PLAT302_ALERT_4_G Anion/Solvent/Minor-Residue Disorder (Resd  6  )       100% Note
PLAT722_ALERT_1_G Angle   Calc      111.00, Rep      109.50 Dev...       1.50 Degree
```

```
             H8A     -C8      -H8B        1_555  1_555  1_555     #   81 Check
PLAT722_ALERT_1_G Angle   Calc      108.00, Rep      109.50 Dev...       1.50 Degree
             N3      -C12     -H12A       1_555  1_555  1_555     #   91 Check
PLAT722_ALERT_1_G Angle   Calc      111.00, Rep      109.50 Dev...       1.50 Degree
             H12B    -C12     -H12C       1_555  1_555  1_555     #   96 Check
PLAT722_ALERT_1_G Angle   Calc      108.00, Rep      109.50 Dev...       1.50 Degree
             N3      -C13     -H13A       1_555  1_555  1_555     #   97 Check
PLAT722_ALERT_1_G Angle   Calc      111.00, Rep      109.50 Dev...       1.50 Degree
             H13B    -C13     -H13C       1_555  1_555  1_555     #  102 Check
PLAT780_ALERT_1_G Coordinates do not Form a Properly Connected Set     Please Do !
PLAT789_ALERT_4_G Atoms with Negative _atom_site_disorder_group  #         24 Check
PLAT794_ALERT_5_G Tentative Bond Valency for Sn1       (IV)      .       3.44 Info
PLAT912_ALERT_4_G Missing # of FCF Reflections Above STh/L=  0.600        344 Note
PLAT913_ALERT_3_G Missing # of Very Strong Reflections in FCF ....          1 Note
PLAT915_ALERT_3_G No Flack x Check Done: Low Friedel Pair Coverage         69 %
PLAT933_ALERT_2_G Number of HKL-OMIT Records in Embedded .res File          4 Note
PLAT941_ALERT_3_G Average HKL Measurement Multiplicity ...........        4.4 Low
PLAT951_ALERT_5_G Calculated (ThMax) and CIF-Reported Kmax Differ           2 Units
PLAT961_ALERT_5_G Dataset Contains no Negative Intensities .......     Please Check
```

---

0 **ALERT level A** = Most likely a serious problem - resolve or explain
0 **ALERT level B** = A potentially serious problem, consider carefully
10 **ALERT level C** = Check. Ensure it is not caused by an omission or oversight
49 **ALERT level G** = General information/check it is not something unexpected

8 ALERT type 1 CIF construction/syntax error, inconsistent or missing data
7 ALERT type 2 Indicator that the structure model may be wrong or deficient
7 ALERT type 3 Indicator that the structure quality may be low
33 ALERT type 4 Improvement, methodology, query or suggestion
4 ALERT type 5 Informative message, check

Datablock: 2

Bond precision: C-C = 0.0085 A Wavelength=0.71073

Cell:	a=9.6792(2)	b=9.6792(2)	c=25.5585(8)
	alpha=90	beta=90	gamma=90
Temperature:	150 K		

	Calculated	Reported
Volume	2394.50(12)	2394.50(12)
Space group	P 43 21 2	P 43 21 2
Hall group	P 4nw 2abw	P 4nw 2abw
Moiety formula	Br6 Sn, 2(C6 H8 N), I2	?
Sum formula	C12 H16 Br6 I2 N2 Sn	C12 H16 Br6 I2 N2 Sn
Mr	1040.18	1040.22
Dx,g cm-3	2.885	2.885
Z	4	4
Mu (mm-1)	13.657	13.658
F000	1872.0	1872.0
F000'	1860.27	
h,k,lmax	13,13,34	12,11,31
Nref	3210[1934]	2724
Tmin,Tmax	0.098,0.505	0.478,1.000
Tmin'	0.057	

Correction method= # Reported T Limits: Tmin=0.478 Tmax=1.000
AbsCorr = MULTI-SCAN

Data completeness= 1.41/0.85   Theta(max)= 29.072

R(reflections)= 0.0254(2439)   wR2(reflections)= 0.0359(2724)

S = 0.982   Npar= 106

The following ALERTS were generated. Each ALERT has the format
test-name_ALERT_alert-type_alert-level.
Click on the hyperlinks for more details of the test.

Alert level C

```
CRYSC01_ALERT_1_C The word below has not been recognised as a standard
            identifier.
            block
CRYSC01_ALERT_1_C No recognised colour has been given for crystal colour.
PLAT048_ALERT_1_C MoietyFormula Not Given (or Incomplete) ........     Please Check
PLAT342_ALERT_3_C Low Bond Precision on  C-C Bonds ..............     0.0085 Ang.
PLAT911_ALERT_3_C Missing FCF Refl Between Thmin & STh/L=    0.600          2 Report
PLAT915_ALERT_3_C No Flack x Check Done: Low Friedel Pair Coverage         79 %
```

Alert level G

```
PLAT434_ALERT_2_G Short Inter HL..HL Contact I1       ..Br3      .       3.22 Ang.
                                                      x,y,z  =      1_555 Check
PLAT790_ALERT_4_G Centre of Gravity not Within Unit Cell: Resd.  #          3 Note
             I2
PLAT794_ALERT_5_G Tentative Bond Valency for Sn1       (IV)      .       3.51 Info
PLAT912_ALERT_4_G Missing # of FCF Reflections Above STh/L=  0.600        166 Note
```

```
PLAT913_ALERT_3_G Missing # of Very Strong Reflections in FCF ....          2 Note
PLAT933_ALERT_2_G Number of HKL-OMIT Records in Embedded .res File          2 Note
PLAT941_ALERT_3_G Average HKL Measurement Multiplicity ..........         4.0 Low
PLAT951_ALERT_5_G Calculated (ThMax) and CIF-Reported Kmax Differ           2 Units
PLAT952_ALERT_5_G Calculated (ThMax) and CIF-Reported Lmax Differ.          3 Units
PLAT958_ALERT_1_G Calculated (ThMax) and Actual (FCF) Lmax Differ.          3 Units
PLAT961_ALERT_5_G Dataset Contains no Negative Intensities .......     Please Check
PLAT978_ALERT_2_G Number C-C Bonds with Positive Residual Density.          0 Info
```

---

0 **ALERT level A** = Most likely a serious problem - resolve or explain
0 **ALERT level B** = A potentially serious problem, consider carefully
6 **ALERT level C** = Check. Ensure it is not caused by an omission or oversight
12 **ALERT level G** = General information/check it is not something unexpected

4 ALERT type 1 CIF construction/syntax error, inconsistent or missing data
3 ALERT type 2 Indicator that the structure model may be wrong or deficient
5 ALERT type 3 Indicator that the structure quality may be low
2 ALERT type 4 Improvement, methodology, query or suggestion
4 ALERT type 5 Informative message, check

Datablock: 3

Bond precision: C-C = 0.0068 A Wavelength=0.71073

Cell:	a=12.8268(7)	b=14.5857(7)	c=13.1741(6)
	alpha=90	beta=107.082(5)	gamma=90
Temperature:	150 K		

	Calculated	Reported
Volume	2356.0(2)	2356.0(2)
Space group	P 21/n	P 21/n
Hall group	-P 2yn	-P 2yn
Moiety formula	Br6 Sn, 2(C6 H8 N), I2	?
Sum formula	C12 H16 Br6 I2 N2 Sn	C12 H16 Br6 I2 N2 Sn
Mr	1040.18	1040.22
Dx,g cm-3	2.932	2.933
Z	4	4
Mu (mm-1)	13.881	13.881
F000	1872.0	1872.0
F000’	1860.27	
h,k,lmax	17,19,18	17,19,17
Nref	6337	5237
Tmin,Tmax	0.050,0.062	0.759,1.000
Tmin’	0.022	

```
Correction method= # Reported T Limits: Tmin=0.759 Tmax=1.000
AbsCorr = MULTI-SCAN

Data completeness= 0.826          Theta(max)= 29.123

                                                wR2(reflections)=
R(reflections)= 0.0313( 4421)                   0.0654( 5237)

S = 1.110                   Npar= 208
```

The following ALERTS were generated. Each ALERT has the format
test-name_ALERT_alert-type_alert-level.
Click on the hyperlinks for more details of the test.

Alert level C

```
PLAT048_ALERT_1_C MoietyFormula Not Given (or Incomplete) ........     Please Check
PLAT420_ALERT_2_C D-H Bond Without Acceptor  N1       --H1A      .     Please Check
PLAT906_ALERT_3_C Large K Value in the Analysis of Variance ......      3.142 Check
PLAT911_ALERT_3_C Missing FCF Refl Between Thmin & STh/L=    0.600         19 Report
PLAT913_ALERT_3_C Missing # of Very Strong Reflections in FCF ....         18 Note
PLAT934_ALERT_3_C Number of (Iobs-Icalc)/Sigma(W) > 10 Outliers ..          1 Check
PLAT977_ALERT_2_C Check Negative Difference Density on H6        .      -0.32 eA-3
PLAT977_ALERT_2_C Check Negative Difference Density on H9A       .      -0.43 eA-3
PLAT977_ALERT_2_C Check Negative Difference Density on H12       .      -0.39 eA-3
```

Alert level G

```
PLAT007_ALERT_5_G Number of Unrefined Donor-H Atoms .............           2 Report
PLAT434_ALERT_2_G Short Inter HL..HL Contact I1       ..Br1      .       3.41 Ang.
                                                    x,y,z  =      1_555 Check
PLAT434_ALERT_2_G Short Inter HL..HL Contact I2       ..Br4      .       3.43 Ang.
                                                  x,1+y,z  =      1_565 Check
PLAT794_ALERT_5_G Tentative Bond Valency for Sn1       (IV)      .       3.50 Info
PLAT912_ALERT_4_G Missing # of FCF Reflections Above STh/L=  0.600       1078 Note
PLAT933_ALERT_2_G Number of HKL-OMIT Records in Embedded .res File         20 Note
PLAT941_ALERT_3_G Average HKL Measurement Multiplicity ..........         2.1 Low
PLAT961_ALERT_5_G Dataset Contains no Negative Intensities .......     Please Check
PLAT978_ALERT_2_G Number C-C Bonds with Positive Residual Density.          0 Info
```

---

0 **ALERT level A** = Most likely a serious problem - resolve or explain
0 **ALERT level B** = A potentially serious problem, consider carefully
9 **ALERT level C** = Check. Ensure it is not caused by an omission or oversight
9 **ALERT level G** = General information/check it is not something unexpected

1 ALERT type 1 CIF construction/syntax error, inconsistent or missing data
8 ALERT type 2 Indicator that the structure model may be wrong or deficient
5 ALERT type 3 Indicator that the structure quality may be low
1 ALERT type 4 Improvement, methodology, query or suggestion
3 ALERT type 5 Informative message, check

---

checkCIF publication errors

Alert level A

```
PUBL004_ALERT_1_A  The contact author's name and address are missing,
            _publ_contact_author_name and _publ_contact_author_address.
PUBL005_ALERT_1_A  _publ_contact_author_email, _publ_contact_author_fax and
            _publ_contact_author_phone are all missing.
            At least one of these should be present.
PUBL006_ALERT_1_A  _publ_requested_journal is missing
            e.g. 'Acta Crystallographica Section C'
PUBL008_ALERT_1_A  _publ_section_title is missing. Title of paper.
PUBL009_ALERT_1_A  _publ_author_name is missing. List of author(s) name(s).
PUBL010_ALERT_1_A  _publ_author_address is missing. Author(s) address(es).
PUBL012_ALERT_1_A  _publ_section_abstract is missing.
            Abstract of paper in English.
```

Alert level G

```
PUBL017_ALERT_1_G The _publ_section_references section is missing or
            empty.
```

---

7 **ALERT level A** = Data missing that is essential or data in wrong format
1 **ALERT level G** = General alerts. Data that may be required is missing

---

Publication of your CIF

You should attempt to resolve as many as possible of the alerts in all categories. Often the minor alerts point to easily fixed oversights, errors and omissions in your CIF or refinement strategy, so attention to these fine details can be worthwhile. In order to resolve some of the more serious problems it may be necessary to carry out additional measurements or structure refinements. However, the nature of your study may justify the reported deviations from journal submission requirements and the more serious of these should be commented upon in the discussion or experimental section of a paper or in the "special_details" fields of the CIF. *checkCIF* was carefully designed to identify outliers and unusual parameters, but every test has its limitations and alerts that are not important in a particular case may appear. Conversely, the absence of alerts does not guarantee there are no aspects of the results needing attention. It is up to the individual to critically assess their own results and, if necessary, seek expert advice.

If level A alerts remain, which you believe to be justified deviations, and you intend to submit this CIF for publication in a journal, you should additionally insert an explanation in your CIF using the Validation Reply Form (VRF) below. This will allow your explanation to be considered as part of the review process.

Validation response form

Please find below a validation response form (VRF) that can be filled in and pasted into your CIF.

```
# start Validation Reply Form
_vrf_PUBL004_GLOBAL
;
PROBLEM: The contact author's name and address are missing,
RESPONSE: ...
;
_vrf_PUBL005_GLOBAL
;
PROBLEM: _publ_contact_author_email, _publ_contact_author_fax and
RESPONSE: ...
;
_vrf_PUBL006_GLOBAL
;
PROBLEM: _publ_requested_journal is missing
RESPONSE: ...
;
_vrf_PUBL008_GLOBAL
;
PROBLEM: _publ_section_title is missing. Title of paper.
RESPONSE: ...
;
_vrf_PUBL009_GLOBAL
;
PROBLEM: _publ_author_name is missing. List of author(s) name(s).
RESPONSE: ...
;
_vrf_PUBL010_GLOBAL
;
PROBLEM: _publ_author_address is missing. Author(s) address(es).
RESPONSE: ...
;
_vrf_PUBL012_GLOBAL
;
PROBLEM: _publ_section_abstract is missing.
RESPONSE: ...
;
# end Validation Reply Form
```

If you wish to submit your CIF for publication in Acta Crystallographica Section C or E, you should upload your CIF via the web. If you wish to submit your CIF for publication in IUCrData you should upload your CIF via the web. If your CIF is to form part of a submission to another IUCr journal, you will be asked, either during electronic submission or by the Co-editor handling your paper, to upload your CIF via our web site.

PLATON version of 19/02/2022; check.def file version of 19/01/2022

Datablock 1 - ellipsoid plot

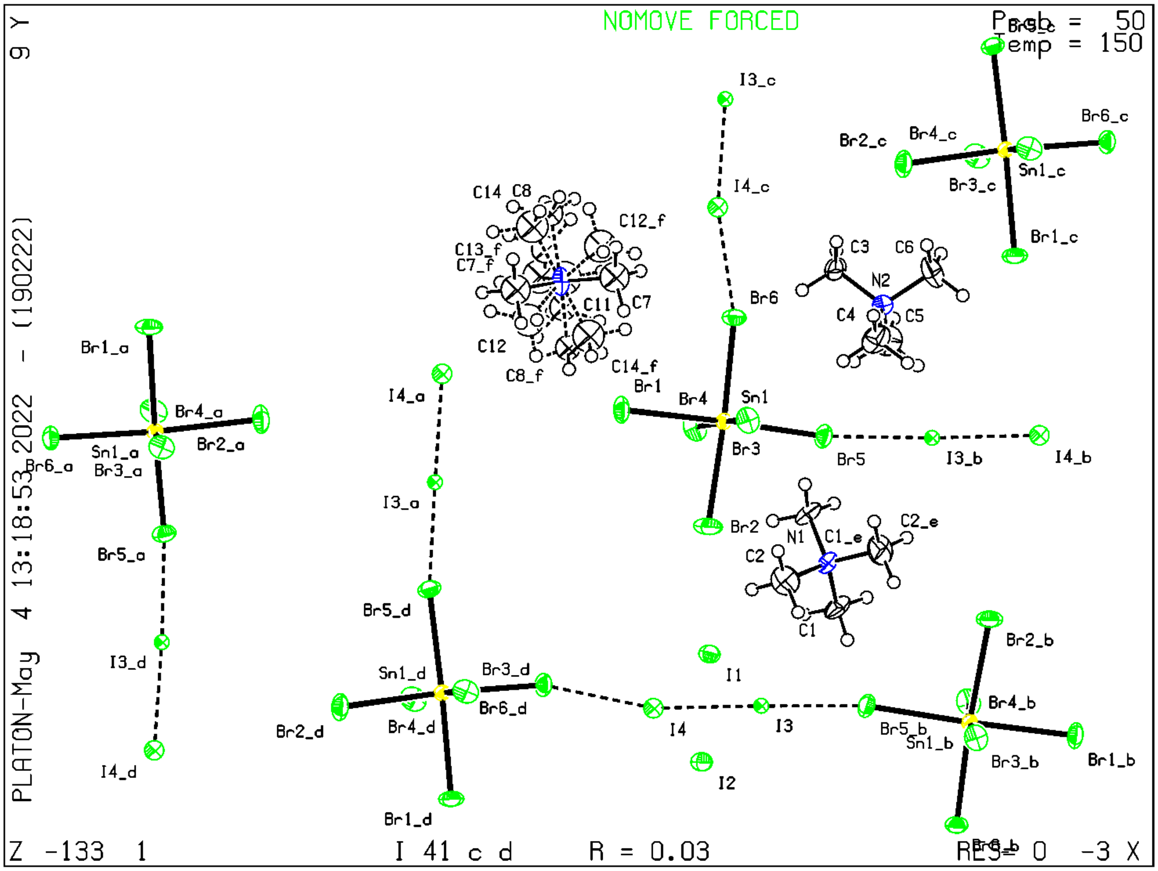

Datablock 2 - ellipsoid plot

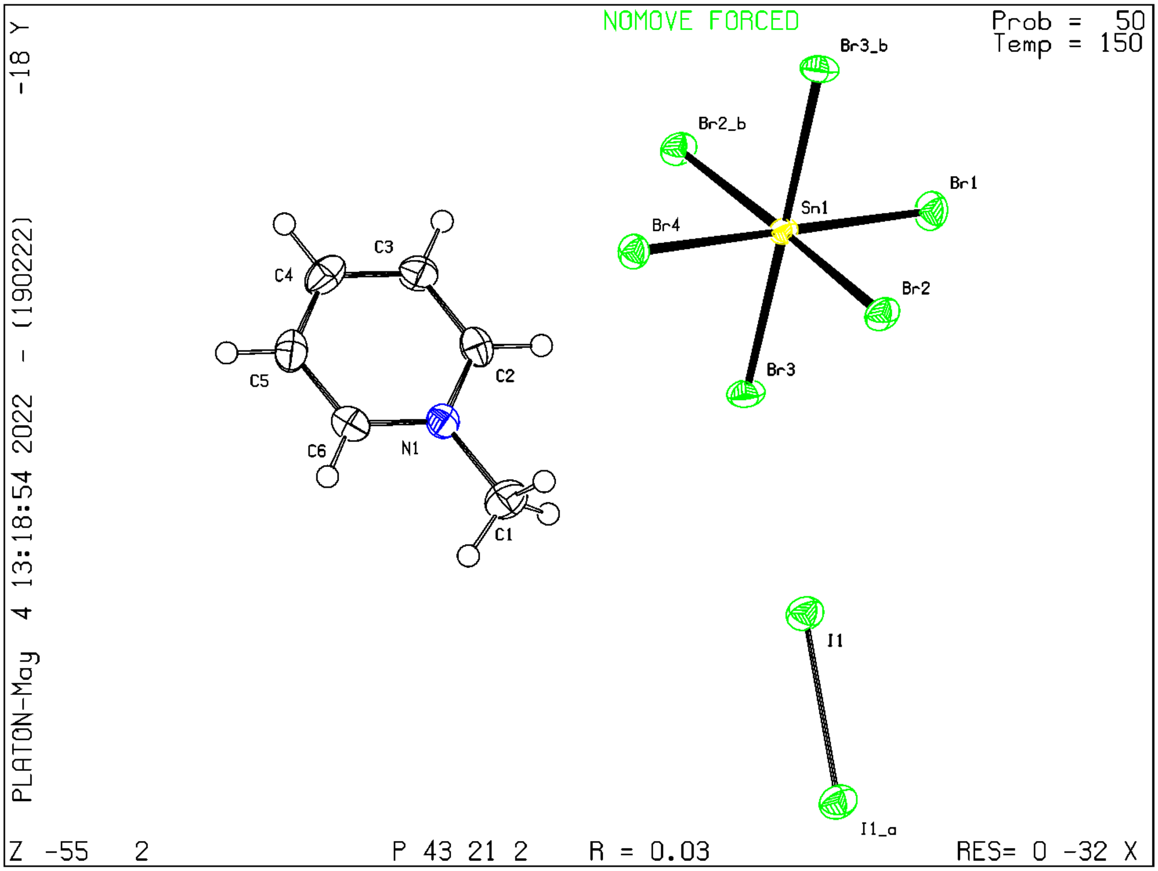

Datablock 3 - ellipsoid plot

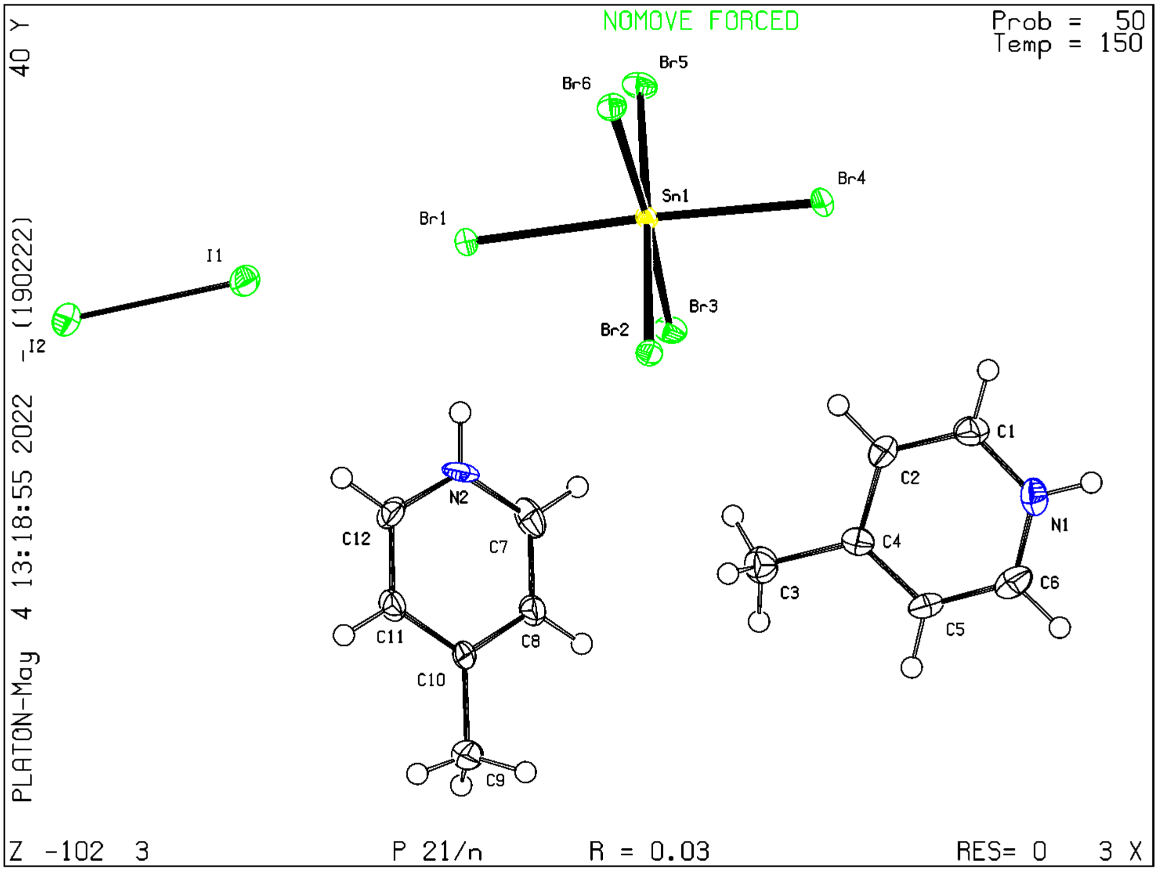

Supplement: Supplementary file 1 [file molecules-27-03859-s001.zip › checkcif.pdf]
