# Supplementary material for: Supramolecular Diiodine-Bromostannate(IV) Complexes: Narrow Bandgap Semiconductors
Source: Molecules. 2022 Jun 16;27(12):3859. doi: 10.3390/molecules27123859 (PMC9229721; doi:10.3390/molecules27123859)
Supplement: Supplementary file 1 [file molecules-27-03859-s001.zip › SI 06.06.2022.pdf]

Supplementary Information

Supramolecular diiodine-bromostannate(IV) complexes: narrow bandgap semiconductors

Nikita A. Korobeynikov, Andrey N. Usoltsev, Pavel A. Abramov, Maxim N. Sokolov and Sergey A. Adonin*

Table S1. Experimental details

	**1**	**2**	**3**
Chemical formula	$C_8H_{24}Br_6I_2N_2Sn$	$C_{12}H_{16}Br_6I_2N_2Sn$	$C_{12}H_{16}Br_6I_2N_2Sn$
M_r	1000,23	1040.22	1040.22
Crystal system, space group	Tetragonal, $I4_1cd$	Tetragonal, $P4_32_12$	Monoclinic, $P2_1/n$
a, b, c (Å)	19.4249 (4), 19.4249 (4), 26.0612 (8)	9.6792 (2), 9.6792 (2), 25.5585 (8)	12.8268 (7), 14.5857 (7), 13.1741 (6)
α, β,γ (°)	90, 90, 90	90, 90, 90	90, 107.082 (5), 90
V (Å^3)	9833.6 (5)	2394.50 (12)	2356.0 (2)
Z	16	4	4
μ (mm^{-1})	13.30	13.66	13.88
Crystal size (mm)	0.10 × 0.10 × 0.07	0.20 × 0.15 × 0.05	0.25 × 0.20 × 0.20
T_{min}, T_{max}	0.661, 1.000	0.478, 1.000	0.759, 1.000
No. of measured, independent and observed [$I > 2\sigma(I)$] reflections	13180, 5187, 4466	6889, 2724, 2439	10940, 5237, 4421
R_{int}	0.029	0.029	0.025
θ values (°)	θ_{max} = 29.0, θ_{min} = 2.1	θ_{max} = 29.1, θ_{min} = 2.3	θ_{max} = 29.1, θ_{min} = 2.0
$(\sin \theta/\lambda)_{max}$ (Å^{-1})	0.682	0.684	0.685
Range of h, k, l	$-25 \le h \le 19$ $-24 \le k \le 15$ $-34 \le l \le 32$	$-12 \le h \le 8$ $-11 \le k \le 8$ $-25 \le l \le 31$	$-17 \le h \le 16$ $-17 \le k \le 19$ $-11 \le l \le 17$
$R[F^2 > 2\sigma(F^2)]$, $wR(F^2)$, S	0.030, 0.071, 1.07	0.025, 0.036, 0.98	0.031, 0.065, 1.11
No. of reflections, parameters, restraints	5183, 216, 1	2724, 106, 0	5237, 208, 0
Weighting scheme	$w = 1/[\sigma^2(F_o^2) + (0.033P)^2]$ where $P = (F_o^2 + 2F_c^2)/3$	$w = 1/[\sigma^2(F_o^2) + (0.0071P)^2]$ where $P = (F_o^2 + 2F_c^2)/3$	$w = 1/[\sigma^2(F_o^2) + (0.0221P)^2]$ where $P = (F_o^2 + 2F_c^2)/3$
$\Delta\rho_{max}$, $\Delta\rho_{min}$ (e Å^{-3})	1.00, -1.02	0.55, -0.56	0.83, -0.96
Absolute structure	Refined as an inversion twin.	Flack x determined using 830 quotients [(I+)-(I-)]/[(I+)+(I-)] (Parsons, Flack and Wagner, Acta Cryst. B69 (2013) 249-259).	–
Absolute structure parameter	0.484 (13)	0.008 (9)	–

Computer programs: *CrysAlis PRO* 1.171.38.41 (Rigaku OD, 2015), *SHELXS2014* (Sheldrick, 2014), *SHELXL2014* (Sheldrick, 2014), ShelXle (Hübschle, 2011), CIFTAB-2014 (Sheldrick, 2014).

Table S2. Selected geometric parameters (Å)

**(1)** main component		**(2)**		**(3)**	
Br1—Sn1	2.6434 (12)	Br1—Sn1	2.5624 (10)	Br1—Sn1	2.6848 (5)
Br2—Sn1	2.6444 (12)	Br2—Sn1	2.5872 (5)	Br2—Sn1	2.5994 (5)
Br3—Sn1	2.5917 (13)	Br3—Sn1	2.6272 (6)	Br3—Sn1	2.5812 (6)
Br4—Sn1	2.5862 (14)	Br4—Sn1	2.6029 (10)	Br4—Sn1	2.6074 (6)
Br5—Sn1	2.5868 (12)	I1—I1^{i}	2.7031 (8)	Br5—Sn1	2.5629 (6)
Br6—Sn1	2.5882 (11)			Br6—Sn1	2.5761 (5)
I1—I2	2.6916 (9)			I1—I2	2.6864 (5)
I3—I4	2.69 (5)				

Symmetry code(s): (i) -*y*, -*x*, -*z*+3/2.

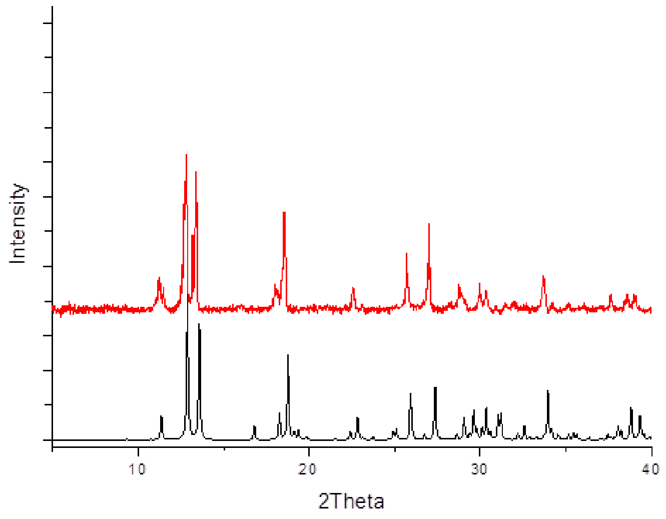

Figure S1. Calculated (*black*) and experimental (*red*) PXRD patterns of **1**

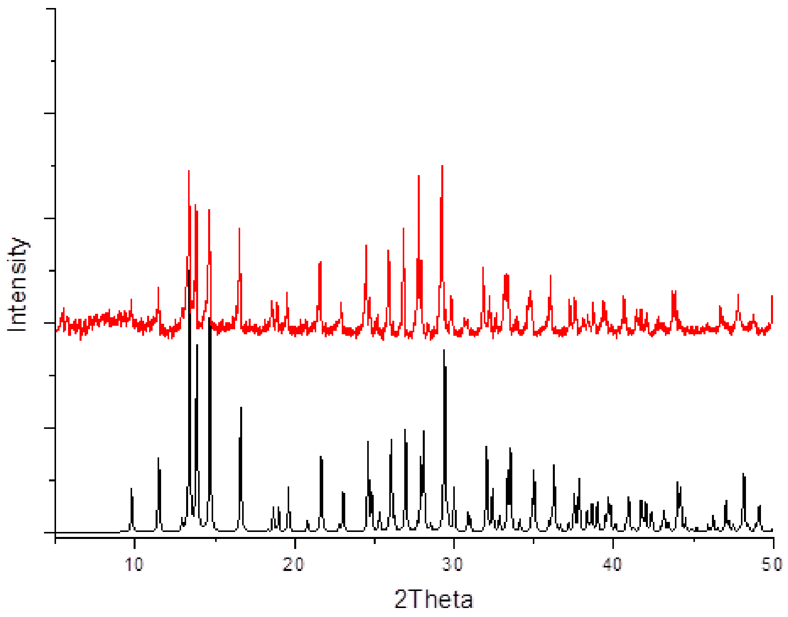

Figure S2. Calculated (*black*) and experimental (*red*) PXRD patterns of **2**

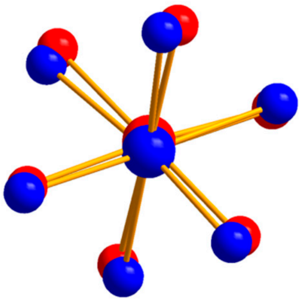

Figure S3. Positional disordering of $[SnBr_6]^{2-}$ octahedron over two closed positions with 0.92 (red) and 0.08 (blue) occupancies.

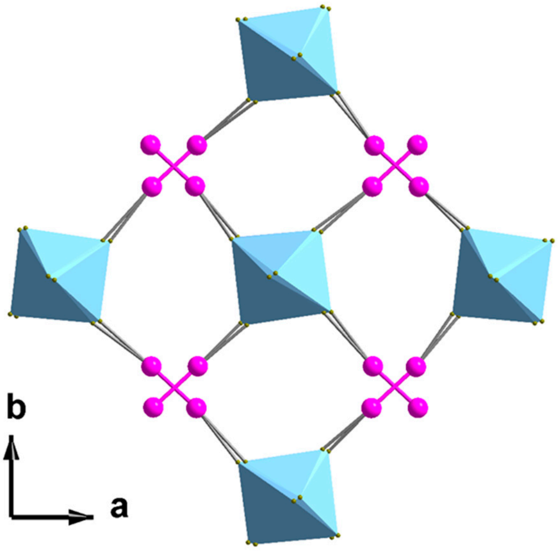

Fig. S4. Structure of the layer formed by $[SnBr_6]^{2-}$ and I_2 units

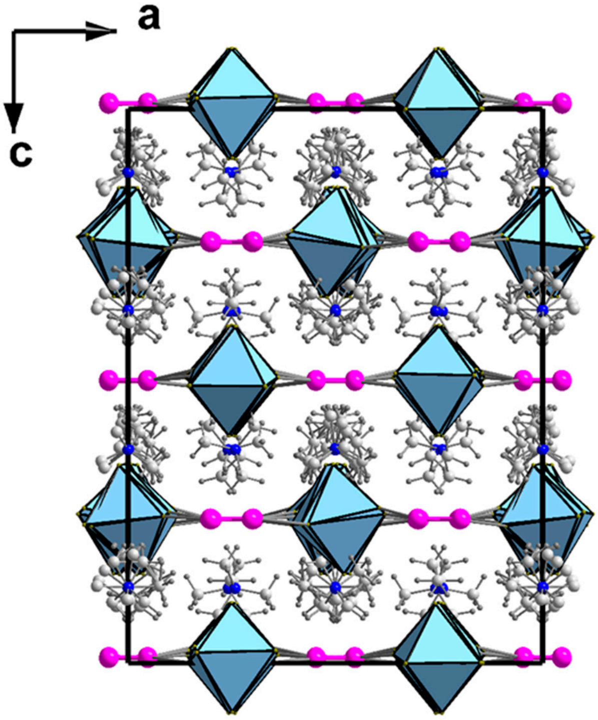

Figure S5. Layered crystal packing of **1**.

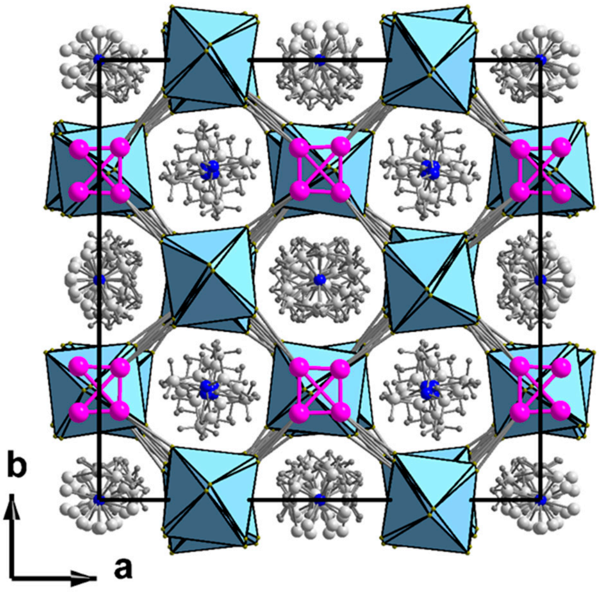

Figure S6. Positions of TMA+ cations in the void between $\{[SnBr_6]^{2-} + I_2\}$ nets

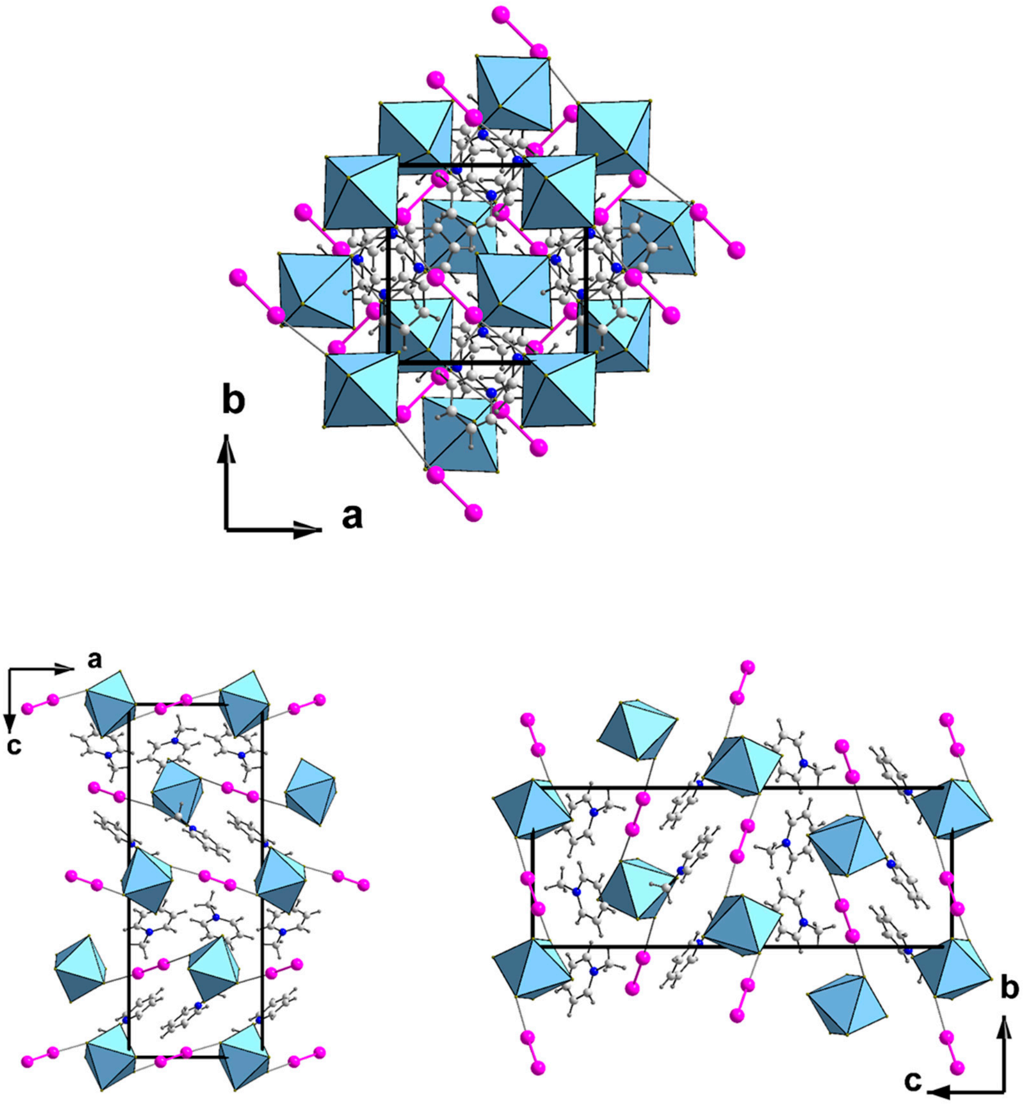

Figure S7. Crystal packing of **2**

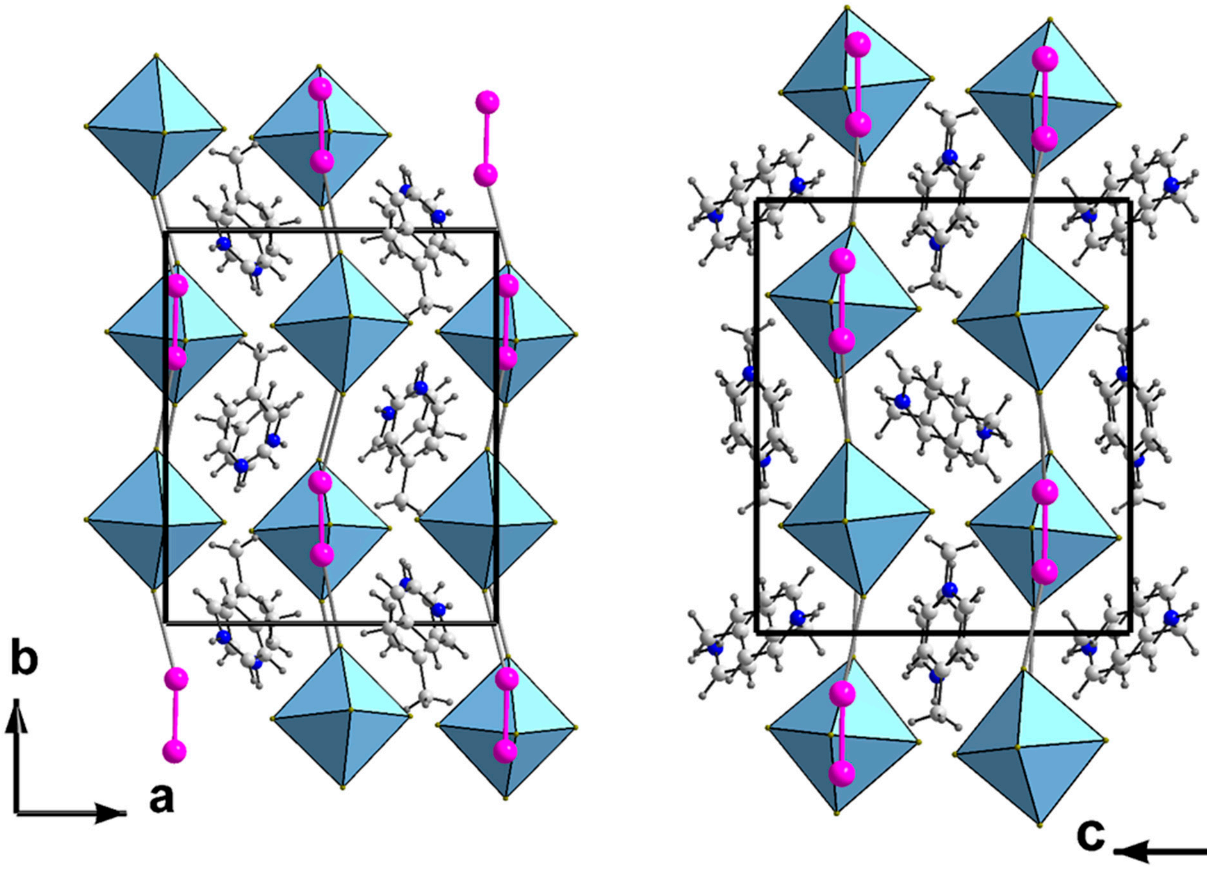

Figure S8. Crystal packing of **3**
